# Prevalence and Factors Associated With Dyslipidemia Among Adults With Type 2 Diabetes: Insights From the National Health and Nutrition Examination Survey (NHANES) 2009-2018

**DOI:** 10.7759/cureus.96124

**Published:** 2025-11-05

**Authors:** Michael U Mochu, Kimberly Osias, Franklin I Nnorom, Oyewale Fakoya, Afolake A Adebayo, Eunice Nathan-Otu, Cynthia E Emanemua

**Affiliations:** 1 Family Medicine, University of Nigeria College of Medicine, Enugu, NGA; 2 Family Medicine, Université Notre Dame D'Haiti, Port au Prince, HTI; 3 Research, Wellstar MCG Health, Augusta, USA; 4 Medicine, Obafemi Awolowo College of Health Science, Olabisi Onabanjo University, Sagamu, NGA; 5 Family Medicine, Nnamdi Azikiwe University, Nnewi, NGA; 6 Medicine and Surgery, Baylor Scott & White Health, Dallas, USA; 7 Medicine, College of Medicine, University of Benin, Benin City, NGA

**Keywords:** cardiovascular risk, dyslipidemia, nhanes, predictors, prevalence, type 2 diabetes

## Abstract

Background and objective

Dyslipidemia is an atherogenic comorbidity common in type 2 diabetes and a major driver of cardiovascular events. This study aimed to examine the prevalence and factors associated with dyslipidemia (sociodemographic and clinical, such as waist circumference, blood pressure, smoking, BMI, and medication status) among U.S. adults with type 2 diabetes based on nationally representative data from the National Health and Nutrition Examination Survey (NHANES) 2009-2018.

Methods

We conducted a cross-sectional analysis of pooled NHANES 2009-2018, including adults aged ≥20 years with type 2 diabetes (n = 14,885; weighted N = 143,669,987). Dyslipidemia was defined by standard lipid thresholds. Survey weights, clusters, and strata were used; survey-adjusted t-tests and Rao-Scott F-tests were employed to compare groups. Multivariable survey logistic regression was utilized to estimate adjusted odds ratios (aOR) for key sociodemographic and clinical factors.

Results

Dyslipidemia was highly prevalent among adults with type 2 diabetes. Adjusted models showed older age (aOR per year: 1.01; p<0.001), female sex (aOR: 1.61; 95% confidence interval (CI): 1.44-1.79), greater waist circumference (aOR per cm: 1.03; 95% CI: 1.02-1.03), higher diastolic blood pressure (BP) (aOR per mmHg: 1.02; 95% CI: 1.01-1.02), and current smoking (aOR 1.34; 95% CI 1.17-1.53) were associated with higher odds. BMI and systolic BP were not significant. Non-Hispanic White and Non-Hispanic Black adults had lower adjusted odds than Mexican Americans.

Conclusions

Dyslipidemia remains a common comorbidity in adults with type 2 diabetes, shaped by both biological and social factors. Targeted, multidimensional interventions are required to address these disparities and reduce cardiovascular risk in this vulnerable population.

## Introduction

Type 2 diabetes mellitus (T2DM) has emerged as a major global health concern, with its prevalence increasing due to lifestyle changes, urbanization, and demographic shifts [1]. T2DM is a serious condition that impacts more than 37 million people in the United States, with the majority being adults, and is strongly associated with high rates of morbidity, mortality, and substantial economic burden [2-3]. In addition to influencing glucose metabolism, T2DM is linked to a wide range of metabolic pathologic alterations, among which dyslipidemia is one of the most common and significant [4]. Dyslipidemia, or the unnatural presence of lipids or lipoproteins in the blood, is a major predisposing factor to cardiovascular disease (CVD), the number one killer of diabetic people [5].

The dyslipidemic pattern typical of T2DM, also called diabetic dyslipidemia, is characterized by increased triglycerides, decreased high-density lipoprotein cholesterol (HDL-C), and small, dense low-density lipoprotein particles (LDL) [6-7]. This lipid pattern is atherogenic and is strongly associated with atherosclerosis, and it is directly associated with significant adverse cardiovascular events such as myocardial infarction, stroke, and cardiovascular death [8]. Although diabetes management has significantly improved, dyslipidemia remains poorly controlled in most individuals, further exacerbating the already high burden of cardiovascular dysfunction in this population [9].

Epidemiological research has consistently revealed that dyslipidemia is more likely to occur in people with T2DM than in the general population [10]. However, the pattern and severity of dyslipidemia differ among populations and are influenced by a range of factors, including genetics, lifestyle, and sociodemographic characteristics such as age, sex, race/ethnicity, education, and income [11-12]. Variations in lifestyle, diet quality, healthcare access, and treatment adherence are also believed to contribute to disparities in lipid control among U.S. racial and socioeconomic groups. Prior evidence highlights significant racial and ethnic differences in dyslipidemia patterns [13]. In addition, other factors such as obesity, physical inactivity, and comorbid hypertension contribute to the lipid abnormalities that are observed in the diabetic population [14].

Understanding the sociodemographic factors associated with dyslipidemia in adults with T2DM is important for several reasons. First, it helps identify high-risk populations that may require screening and a more aggressive response [15]. Second, it provides evidence to inform resource allocation and policymaking, particularly in overcoming health inequities [16]. Third, by examining the details of dyslipidemia prevalence and distribution, clinicians and other health practitioners in the community can refine CVD risk stratification and offer customized treatment interventions, including lifestyle changes and pharmacotherapy [17-18].

The National Health and Nutrition Examination Survey (NHANES) offers a valuable means of assessing the health and nutritional status of the U.S. population through the collection and analysis of data across a wide spectrum of health and nutrition topics [19]. As a nationally representative survey, NHANES collects extensive information on health behaviors, laboratory indicators, chronic diseases, and the use of prescription medications, enabling a detailed study of the prevalence of dyslipidemia and its correlates among adults with T2DM in the United States [20]. Given the increasing burden of T2DM and its strong association with dyslipidemia, this study seeks to address an existing knowledge gap by estimating the prevalence of dyslipidemia among adults with T2DM in the U.S. and identifying the sociodemographic factors associated with it. The main objective of the study is to determine the prevalence and sociodemographic and clinical correlates of dyslipidemia in adults with type 2 diabetes.

## Materials and methods

Study design and data source

This study employed a cross-sectional design and used publicly available data from NHANES [21]. NHANES is conducted by the National Center for Health Statistics (NCHS) and utilizes a complex, multistage probability sampling design to generate nationally representative estimates of the non-institutionalized U.S. population. The survey integrates interviews, physical examinations, and laboratory testing, providing a robust dataset for epidemiologic research. For the present analysis, we pooled data from the 2009-2018 NHANES cycles to maximize sample size and statistical power while ensuring consistent laboratory methods for lipid and glucose measurements across cycles.

Study population

The study population involved adults aged 20 years and older who met the criteria for type 2 diabetes. Diabetes status was determined through self-report of a physician diagnosis (NHANES variable DIQ010), use of glucose-lowering medications identified in the NHANES prescription medications file (RXQ_/RXDD files), or laboratory evidence of diabetes based on fasting plasma glucose (LBXGLU) ≥126 mg/dL and glycohemoglobin (HbA1c, LBXGH) ≥6.5%, consistent with standard diagnostic criteria. To better approximate type 2 diabetes and minimize misclassification, individuals diagnosed before age 30 who reported using only insulin were excluded to reduce the likelihood of including those with type 1 diabetes. Pregnant women were excluded due to physiologic alterations in lipid metabolism during pregnancy. Participants lacking data for key lipid variables were also excluded from the final analytic sample.

Variables/measures

The primary outcome was dyslipidemia, defined using established clinical cutoffs for abnormal lipid levels. Laboratory measures included total cholesterol, LDL-C, HDL-C, and triglycerides. Dyslipidemia was defined as the presence of any abnormality, including LDL-C ≥130 mg/dL, HDL-C <40 mg/dL in men or <50 mg/dL in women, triglycerides ≥150 mg/dL, or total cholesterol ≥200 mg/dL, irrespective of reported lipid-lowering medication use. Information on lipid-lowering medication use (e.g., statins) is collected in NHANES prescription medication files; however, for the primary analysis, dyslipidemia status was based on measured lipids. The main independent variables of interest were sociodemographic factors, including age, gender, race/ethnicity, education, marital status, health insurance coverage, and poverty-income ratio.

Education categories (less than high school, high school/GED, college or greater) were defined to match commonly used NHANES groupings and prior population studies. Physical activity was categorized by self-reported days per week of moderate-to-vigorous activity (1-2, 3-4, ≥5 days) consistent with prior NHANES analyses. Income was analyzed using the poverty-income ratio (PIR) as a continuous measure; for categorical analyses, thresholds were selected based on federal poverty guidelines and prior epidemiologic studies. Additional covariates included in the models were BMI, waist circumference, hypertension status, smoking, and physical activity, all known correlates of lipid metabolism and cardiovascular risk. For transparency and reproducibility, the NHANES variable names and items used were obtained from the NHANES analytic codebook (variable names and measure descriptions), which is publicly available on the NCHS website.

Statistical analysis

All analyses accounted for NHANES's complex survey design by incorporating sampling weights, primary sampling units, and strata to produce valid national estimates. For pooled analyses across the five two-year NHANES cycles (2009-2010 through 2017-2018), we recalculated the two-year MEC sample weights as recommended by NCHS guidance by dividing the two-year MEC examination weight by the number of cycles combined (5). We used these recalculated weights together with the recommended strata and PSU variables in all survey procedures. Descriptive statistics were used to summarize the prevalence of dyslipidemia across sociodemographic and clinical subgroups. Because this analysis uses complex survey design data to produce nationally representative estimates, we employed survey-design-based F-tests (Rao-Scott adjusted F-tests) to compare groups for categorical variables and survey-adjusted t-tests for continuous variables.

To examine predictors of dyslipidemia among adults with type 2 diabetes, we constructed multivariable logistic regression models adjusting for sociodemographic and clinical covariates. Multicollinearity was assessed using the variance inflation factor (VIF). Most variables demonstrated low VIF values (<3), with an overall mean VIF of 2.28. BMI and waist circumference showed higher VIF values (7.79 and 7.96, respectively), consistent with their known correlation as measures of adiposity; however, these levels are below the conventional threshold of 10, suggesting no serious multicollinearity concerns. All statistical analyses were performed using Stata version 18 (StataCorp, College Station, TX), and statistical significance was evaluated using two-sided tests with an alpha of 0.05.

Missing data

Given the complex survey design of NHANES, no imputation methods were used to handle missing data. Instead, a complete case analysis was conducted. The proportion of missing data for most analytic variables was low, generally under 10%. For example, BMI had 5.2% missing values, while key lipid measures and most sociodemographic variables exhibited even lower rates of missingness. Such minimal missing data reduces the risk of substantial bias in the estimates.

Ethical considerations

The National Center approved NHANES protocols for the Health Statistics Research Ethics Review Board, and all participants provided informed consent before participation. Since NHANES data are publicly available, de-identified, and ethically approved, no additional institutional review board approval was required for this secondary data analysis.

## Results

Table 1 below presents the weighted descriptive characteristics of 14,885 adults with type 2 diabetes included in the analytic sample, corresponding to a U.S. population estimate of 143,669,987 adults. Characteristics are shown according to dyslipidemia status.

**Table 1:** Weighted sociodemographic and clinical characteristics of adults with and without dyslipidemia, NHANES 2009–2018 –: intentionally left blank Continuous variables are expressed as means with standard deviations and compared using survey-adjusted t-tests. Categorical variables are summarized as weighted frequencies and percentages and compared using Rao–Scott adjusted F-tests. Reported p-values are derived from these survey-adjusted tests NHANES: the National Health and Nutrition Examination Survey; GED: General Educational Development; BP: blood pressure; BMI: body mass index

Characteristic	Dyslipidemia, no (n = 59,635,795)	Dyslipidemia, yes, (n = 84,034,192)	F-test/T-test	P-value
Age in years (mean ± SD)	45.40 ± 18.29	49.27 ± 15.68	t = -8.86	<0.001
BMI in kg/m² (mean ± SD)	27.89 ± 6.65	30.54 ± 6.91	t = -16.58	<0.001
Income-to-poverty ratio (mean ± SD)	2.99 ± 1.67	3.00± 1.65	t = -0.60	0.551
Waist circumference in cm (mean ± SD)	96.47 ± 16.72	103.45 ± 16.09	t = -17.15	<0.001
Mean systolic BP in mmHg (mean ± SD)	120.75 ± 16.92	123.85 ± 16.94	t = -7.88	<0.001
Mean diastolic BP in mmHg (mean ± SD)	69.34 ± 11.80	72.14 ± 11.82	t = -10.43	<0.001
Gender, n (%)	-	-	F = 33.34	<0.001
Male	31,428,370 (45%)	38,553,438 (55%)	-	-
Female	28,207,425 (38%)	45,480,754 (62%)	-	-
Education level, n (%)	-	-	F = 7.33	<0.001
Less than high school	7,547,863 (39%)	11,757,228 (61%)	-	-
High school graduate/GED	12,922,017 (39%)	19,971,180 (61%)	-	-
College+	39,165,914 (43%)	52,305,784 (57%)	-	-
Marital status, n (%)	-	-	F = 62.74	<0.001
Married/partnered	35,969,108 (40%)	54,780,728 (60%)	-	-
Previously married	9,664,290 (36%)	16,910,078 (64%)	-	-
Never married	14,002,397 (53%)	12,343,386 (47%)	-	-
Insurance cover, n (%)	-	-	F = 1.11	0.296
No	9,237,610 (40%)	13,692,564 (60%)	-	-
Yes	50,398185 (42%)	70,341,628 (58%)	-	-
Hypertension, n (%)	-	-	F = 26.47	<0.001
No	42,069,483 (43%)	54,701,516 (57%)	-	-
Yes	17,566,313 (37%)	29,332,676 (63%)	-	-
Smoking, n (%)	-	-	F = 7.17	0.001
Never	34,596,839 (43%)	45,433,772 (57%)	-	-
Former	14,508,773 (40%)	22,085,254 (60%)	-	-
Current	10,530,183 (39%)	16,515,166 (61%)	-	-
Physical activity (moderate to vigorous), n (%)	-	-	F = 0.60	0.538
1-2 days	11,689,542 (42%)	15,883,282 (58%)	-	-
3-4 days	47,933,814 (41%)	68,118,517 (59%)	-	-
≥5 days	12,440 (28%)	32,394 (72%)	-	-
Race/ethnicity, n (%)	-	-	F = 31.32	<0.001
Mexican American	4,764,298 (37%)	8,197,454 (63%)	-	-
Other Hispanic	3,752,427 (40%)	5,703,741 (60%)	-	-
Non-Hispanic White	42,266,781 (41%)	61,844,133 (59%)	-	-
Non-Hispanic Black	8,852,290 (52%)	8,288,865 (48%)	-	-

As shown in Table 1, adults with dyslipidemia were significantly older than those without the condition (49.27 ± 15.68 years vs. 45.40 ± 18.29 years, p<0.001). Mean BMI was higher among adults with dyslipidemia (30.54 ± 6.91 kg/m²) compared to those without dyslipidemia (27.89 ± 6.65 kg/m², p<0.001). Similarly, waist circumference was greater in the dyslipidemia group (103.45 ± 16.09 cm) than in the non-dyslipidemia group (96.47 ± 16.72 cm, p<0.001). Blood pressure measures also differed, with higher systolic (123.85 ± 16.94 mmHg vs. 120.75 ± 16.92 mmHg, p<0.001) and diastolic (72.14 ± 11.82 mmHg vs. 69.34 ± 11.80 mmHg, p<0.001) readings observed in adults with dyslipidemia.

Gender differences were also apparent: among men, 38,553,438 (55%) had dyslipidemia, while 31,428,370 (45%) did not; among women, 45,480,754 (62%) had dyslipidemia compared to 28,207,425 (38%) without the condition (p<0.001). Educational attainment showed gradients: among those with less than high school education, 11,757,228 (61%) had dyslipidemia compared to 7,547,863 (39%) without; for high school graduates or GED holders, 19,971,180 (61%) had dyslipidemia versus 12,922,017 (39%) without; and among those with college education or more, 52,305,784 (57%) had dyslipidemia compared to 39,165,914 (43%) without (p<0.001).

Marital status was also associated with dyslipidemia. Of those married or partnered, 54,780,728 (60%) had dyslipidemia compared to 35,969,108 (40%) without. Among previously married individuals, 16,910,078 (64%) had dyslipidemia compared to 9,664,290 (36%) without. In contrast, among never-married adults, only 12,343,386 (47%) had dyslipidemia compared to 14,002,397 (53%) without (p<0.001).

Insurance coverage was not significantly associated with dyslipidemia (p = 0.296). Among uninsured participants, 13,692,564 (60%) had dyslipidemia compared to 9,237,610 (40%) without. Similarly, among insured participants, 70,341,628 (58%) had dyslipidemia compared to 50,398,185 (42%) without. In contrast, hypertension status was strongly associated with dyslipidemia: among hypertensive adults, 29,332,676 (63%) had dyslipidemia, compared to 17,566,313 (37%) without, whereas among non-hypertensive adults, 54,701,516 (57%) had dyslipidemia, compared to 42,069,483 (43%) without (p<0.001).

Smoking status also showed differences (p = 0.001). Among never smokers, 45,433,772 (57%) had dyslipidemia compared to 34,596,839 (43%) without. Former smokers demonstrated a prevalence of 22,085,254 (60%) with vs. 14,508,773 (40%) without, while current smokers showed the highest prevalence at 16,515,166 (61%) with vs. 10,530,183 (39%) without. Physical activity was not significantly associated with dyslipidemia (p = 0.538), with prevalence estimates of 15,883,282 (58%) for those active one to two days per week, vs. 11,689,542 (42%) without, and 68,118,517 (59%) for those active three to four days per week vs. 47,933,814 (41%) without. Although estimates suggested a prevalence of 32,394 (72%) among those active ≥5 days per week, the unweighted sample size for this group was tiny, compared with 12,440 (28%) for those without.

Significant racial and ethnic differences were observed (p<0.001). Mexican Americans had a dyslipidemia prevalence of 8,197,454 (63%) with vs. 4,764,298 (37%) without, and Other Hispanics had a prevalence of 5,703,741 (60%) with vs. 3,752,427 (40%) without. Among Non-Hispanic Whites, 61,844,133 (59%) had dyslipidemia compared to 42,266,781 (41%) without. In contrast, Non-Hispanic Blacks had the lowest prevalence, with only 8,288,865 (48%) affected compared to 8,852,290 (52%) without dyslipidemia.

Table 2 below presents odds ratios (ORs) with 95% confidence intervals (CIs) from a multivariable logistic regression model assessing sociodemographic and clinical factors associated with dyslipidemia in adults with type 2 diabetes.

**Table 2:** Survey-weighted multivariable logistic regression of factors associated with dyslipidemia among adults with type 2 diabetes, NHANES 2009–2018 –: intentionally left blank All models accounted for the NHANES complex sampling design and adjusted for age, gender, BMI, waist circumference, blood pressure, income-to-poverty ratio, education, marital status, insurance, hypertension, smoking, physical activity, and race/ethnicity. NHANES: the National Health and Nutrition Examination Survey; OR: odds ratio; CI: confidence interval; GED: General Educational Development; BP: blood pressure; BMI: body mass index

Factor	OR (95% CI)	P-value
Age (in years)	1.01 (1.01–1.01)	<0.001
Gender (female vs. male)	1.61 (1.44–1.79)	<0.001
BMI (kg/m²)	1.00 (0.99–1.02)	0.622
Income-to-poverty ratio (IPR)	1.02 (0.99–1.04)	0.123
Waist circumference (cm)	1.03 (1.02–1.03)	<0.001
Mean systolic BP (mmHg)	1.00 (0.996–1.003)	0.837
Mean diastolic BP (mmHg)	1.02 (1.01–1.02)	<0.001
Race/ethnicity	–	–
Other Hispanic vs. Mexican American	0.96 (0.80–1.17)	0.709
Non-Hispanic White vs. Mexican American	0.82 (0.72–0.94)	0.006
Non-Hispanic Black vs. Mexican American	0.53 (0.46–0.61)	<0.001
Education level	–	–
High school/GED vs. those without	1.01 (0.89–1.15)	0.844
College+ vs. those without	0.93 (0.82–1.04)	0.205
Marital status	–	–
Previously married vs. married/partnered	1.01 (0.89–1.15)	0.856
Never married vs. married/partnered	0.77 (0.69–0.86)	<0.001
Insurance cover (yes vs. no)	0.83 (0.73–0.96)	0.010
Hypertension (yes vs. no)	0.85 (0.76–0.94)	0.003
Smoking status	–	–
Former vs. never	1.00 (0.90–1.10)	0.973
Current vs. never	1.34 (1.17–1.53)	<0.001
Physical activity	–	–
3–4 days/week vs. 1–2 days	0.97 (0.86–1.09)	0.598
≥5 days/week vs. 1–2 days	1.61 (0.18–14.42)	0.665

The findings from Table 2 above revealed that increasing age was independently associated with higher odds of dyslipidemia (OR: 1.01; 95% CI: 1.01-1.01; p<0.001), implying that each additional year of age is associated with a slight increase in the likelihood of dyslipidemia. Gender differences were pronounced, with women having 61% higher odds of dyslipidemia compared with men (OR: 1.61; 95% CI: 1.44-1.79; p<0.001). Measures of adiposity revealed a strong effect of waist circumference, where each 1 cm increase was associated with 3% higher odds of dyslipidemia (OR: 1.03; 95% CI: 1.02-1.03; p<0.001). In contrast, BMI was not independently associated with dyslipidemia after adjustment (OR: 1.00; 95% CI: 0.99-1.02; p = 0.622).

Blood pressure associations were mixed. Systolic BP was not significantly related to dyslipidemia (OR: 1.00; 95% CI: 0.996-1.003; p = 0.837), whereas higher diastolic BP was a decisive factor. Each 1 mmHg increase in diastolic BP was associated with 2% higher odds of dyslipidemia (OR: 1.02; 95% CI: 1.01-1.02; p<0.001). Race and ethnicity showed essential disparities. Compared with Mexican Americans, Non-Hispanic Whites (OR: 0.82; 95% CI: 0.72-0.94; p = 0.006) and Non-Hispanic Blacks (OR: 0.53; 95% CI: 0.46-0.61; p<0.001) had significantly lower odds of dyslipidemia. No significant differences were observed for Other Hispanics (OR: 0.96; 95% CI: 0.80-1.17; p = 0.709).

Socioeconomic indicators, including income-to-poverty ratio and education, were not significant factors associated with dyslipidemia in the adjusted model. However, marital status showed an effect: never-married adults had lower odds of dyslipidemia compared with married or partnered individuals (OR: 0.77; 95% CI: 0.69-0.86; p<0.001). Insurance status was inversely associated with dyslipidemia, with insured individuals having lower odds (OR: 0.83; 95% CI: 0.73-0.96; p = 0.010). Similarly, hypertension was associated with reduced odds of dyslipidemia after adjustment (OR: 0.85; 95% CI: 0.76-0.94; p = 0.003), suggesting overlapping treatment or clinical management effects.

Smoking behavior demonstrated a strong influence. Current smokers had 34% higher odds of dyslipidemia compared to never smokers (OR: 1.34; 95% CI: 1.17-1.53; p<0.001). Former smokers did not differ significantly from never smokers (OR: 1.00; 95% CI: 0.90-1.10; p = 0.973). Finally, physical activity was not significantly related to dyslipidemia. Compared to individuals active one to two days per week, neither those active three to four days per week (OR: 0.97; 95% CI: 0.86-1.09; p = 0.598) nor those active ≥5 days per week (OR: 1.61; 95% CI: 0.18-14.42; p = 0.665) demonstrated significant differences in dyslipidemia prevalence.

## Discussion

This study provides nationally representative evidence on the prevalence and factors associated with dyslipidemia among adults with type 2 diabetes in the United States using NHANES 2009-2018 data. Several important sociodemographic and clinical characteristics were identified as significant factors. Age, female gender, central obesity as measured by waist circumference, and elevated diastolic BP were all strongly associated with higher odds of dyslipidemia. These findings are consistent with prior studies showing that aging and obesity exacerbate lipid abnormalities in diabetes through metabolic and hormonal pathways [4,7,14]. The strong association between waist circumference and dyslipidemia reinforces the role of central adiposity as a more reliable marker of metabolic risk than BMI, which was not significant in this study, reflecting their known collinearity and differential predictive ability [6,13]. Neville et al. demonstrated that waist circumference is a superior anthropometric marker for predicting cardiometabolic risk compared with BMI, as it better reflects central adiposity and visceral fat accumulation and more accurately detects changes associated with cardiovascular and metabolic disease [22].

Racial and ethnic differences in lipid patterns have also been observed, arising from a complex interplay of genetic, dietary, and cultural influences. For instance, non-Hispanic Blacks generally exhibit lower triglyceride levels and higher HDL cholesterol than non-Hispanic Whites, while Hispanics often present with higher triglycerides and lower HDL levels; Asians may develop dyslipidemia at lower body weights due to variations in fat and insulin metabolism [13,20]. Socioeconomic position, as measured by income-to-poverty ratio and education, was not significantly associated with dyslipidemia after adjustment, suggesting that clinical factors and lifestyle behaviors may have a greater influence. However, marital status and smoking behavior demonstrated clear associations: never-married individuals and current smokers were at higher risk, pointing to the interplay of social determinants and health behaviors in shaping cardiometabolic risk.

Clinical covariates further highlighted modifiable factors. Individuals with health insurance and those with comorbid hypertension had lower odds of dyslipidemia (adjusted OR <1), potentially reflecting greater access to screening, treatment, and integrated care. The inverse association between self-reported hypertension and measured dyslipidemia (adjusted OR <1) may reflect treatment and health-care engagement: individuals with diagnosed hypertension are more likely to be under medical care and to receive lipid-lowering medications, which can lower measured lipid levels. Residual confounding by medication use or differences in care access may therefore account for this finding.

Conversely, current smoking substantially increased dyslipidemia risk, consistent with the adverse lipid effects of tobacco use [5,18]. This is because smoking behavior remains another crucial factor; current smokers, particularly those who are younger, have higher HbA1c levels, or reside in rural areas, are more likely to exhibit metabolic dyslipidemia characterized by elevated triglycerides and reduced HDL cholesterol [9,18]. Together, these observations reinforce the notion that effective dyslipidemia control in adults with type 2 diabetes requires multidimensional strategies that address biological, behavioral, and sociocultural determinants of lipid metabolism. Physical activity did not show an apparent protective effect, which may be due to self-report bias or insufficient intensity of activity captured by NHANES measures.

Overall, these findings emphasize the multifactorial nature of dyslipidemia in adults with type 2 diabetes, shaped by biological, behavioral, and social factors. They highlight the need for a comprehensive risk assessment that incorporates central adiposity, blood pressure control, smoking status, and social determinants in addition to traditional demographic variables.

Strengths and limitations

This study’s main strength lies in its use of NHANES, a large, nationally representative dataset with standardized data collection protocols, enabling findings to be generalized to the U.S. adult population with diabetes. The inclusion of a broad range of sociodemographic and clinical covariates further enhances the robustness of the analysis. However, several limitations should be acknowledged. The cross-sectional design precludes causal inference, and reliance on self-reported measures (such as smoking and physical activity) may introduce bias. Additionally, missing data led to a complete case analysis, which may have reduced the sample size and introduced bias if missingness was not random.

Dyslipidemia was defined by measured lipid thresholds without adjusting the definition for current lipid-lowering therapy. Because participants on statins or other lipid-lowering medications may have lower measured lipid levels, our approach could lead to an underestimation of the true prevalence of dyslipidemia. Lack of adjustment for medication is therefore a potential source of misclassification; the direction of bias is likely toward the null for associations with some factors. Additional limitations include reliance on the NHANES fasting subsample for certain lipid measures: LDL-C in NHANES is commonly estimated using the Friedewald equation (which may be inaccurate when triglycerides are elevated), and the fasting subsample may not be fully representative of all participants. These factors could introduce selection bias and measurement error. Further, unmeasured confounders such as diabetes duration, dietary intake, and medication adherence were not available for adjustment and may influence observed associations.

The observed inverse association between self-reported hypertension and dyslipidemia (adjusted OR <1) may reflect treatment and care-seeking differences: persons with diagnosed hypertension are often more engaged in clinical care and more likely to be prescribed lipid-lowering therapy, which could lower measured lipid values. Residual confounding (for example, differences in medication use, diabetes duration, or healthcare access) may also explain this counterintuitive finding. Because of the cross-sectional design, we cannot determine whether treatment or other factors account for this association; longitudinal data or explicit adjustment for medication use would be needed.

Future research directions

Future analyses should incorporate medication use (for example, by classifying treated participants with historically elevated lipids as having dyslipidemia or by including medication use as a covariate). Additionally, they should apply longitudinal designs to better clarify causal relationships between sociodemographic and clinical factors associated with dyslipidemia. Statistical techniques such as multiple imputation should be used to more effectively address missing data and minimize bias. We did not explicitly model temporal variation across NHANES cycles (survey year/cycle indicator). Unmeasured temporal trends in treatment patterns (e.g., increasing statin use over time) might affect prevalence and associations and should be examined in future sensitivity analyses. Further work is also needed to examine genetic, lifestyle, and treatment-related contributors to disparities in lipid control among adults with type 2 diabetes.

## Conclusions

In this nationally representative analysis of U.S. adults with type 2 diabetes, dyslipidemia was highly prevalent and strongly influenced by age, sex, central adiposity, BP, smoking status, and social factors such as marital status and insurance coverage. Racial and ethnic differences were also evident, underscoring the role of population-level disparities in shaping lipid risk. These findings highlight the need for targeted interventions that go beyond glycemic control to address obesity, lifestyle behaviors, and social determinants of health in reducing the burden of dyslipidemia and its cardiovascular consequences among people living with diabetes.
